# Sustained pain-related depression of behavior: effects of intraplantar formalin and complete freund’s adjuvant on intracranial self-stimulation (ICSS) and endogenous kappa opioid biomarkers in rats

**DOI:** 10.1186/1744-8069-10-62

**Published:** 2014-09-23

**Authors:** Michael D Leitl, David N Potter, Kejun Cheng, Kenner C Rice, William A Carlezon, S Stevens Negus

**Affiliations:** Department of Pharmacology and Toxicology, Virginia Commonwealth University, 410 N. 12th St., PO Box 980613, Richmond, VA USA; Behavioral Genetics Laboratory, McLean Hospital, Harvard Medical School, Belmont, MA USA; Chemical Biology Research Branch, National Institute on Drug Abuse and National Institute on Alcohol Abuse and Alcoholism, National Institutes of Health, Bethesda, MD USA

**Keywords:** Complete Freund’s adjuvant, Formalin, Pain/analgesics, Depression, Intracranial self-stimulation, Kappa opioid

## Abstract

**Background:**

Intraplantar administration of complete Freund's adjuvant (CFA) and formalin are two noxious stimuli commonly used to produce sustained pain-related behaviors in rodents for research on neurobiology and treatment of pain. One clinically relevant manifestation of pain is depression of behavior and mood. This study compared effects of intraplantar CFA and formalin on depression of positively reinforced operant behavior in an assay of intracranial self-stimulation (ICSS) in rats. Effects of CFA and formalin on other physiological and behavioral measures, and opioid effects on formalin-induced depression of ICSS, were also examined.

**Results:**

There were four main findings. First, consistent with previous studies, both CFA and formalin produced similar paw swelling and mechanical hypersensitivity. Second, CFA produced weak and transient depression of ICSS, whereas formalin produced a more robust and sustained depression of ICSS that lasted at least 14 days. Third, formalin-induced depression of ICSS was reversed by morphine doses that did not significantly alter ICSS in saline-treated rats, suggesting that formalin effects on ICSS can be interpreted as an example of pain-related and analgesic-reversible depression of behavior. Finally, formalin-induced depression of ICSS was not associated with changes in central biomarkers for activation of endogenous kappa opioid systems, which have been implicated in depressive-like states in rodents, nor was it blocked by the kappa antagonist norbinaltorphimine.

**Conclusions:**

These results suggest differential efficacy of sustained pain stimuli to depress brain reward function in rats as assessed with ICSS. Formalin-induced depression of ICSS does not appear to engage brain kappa opioid systems.

**Electronic supplementary material:**

The online version of this article (doi:10.1186/1744-8069-10-62) contains supplementary material, which is available to authorized users.

## Introduction

Preclinical procedures to evaluate pain and analgesia in laboratory animals play a key role in research on both neurobiology of pain and analgesic drug development [1–3]. Two common chemical challenges for induction of sustained pain states in rodents are intraplantar administration of Complete Freund’s Adjuvant (CFA) and formalin. CFA is a heat-killed bacterial suspension that elicits an immune response at the site of its injection. For example, CFA administration in the hindpaw of rats or mice produces paw edema [4–6] and increased local concentrations of inflammatory cytokines and trophic factors [7]. These and other inflammatory mediators contribute to peripheral and central sensitization of nociceptive neural pathways [8–10], and this neural hypersensitivity correlates with a sustained behavioral hypersensitivity of withdrawal responses to mechanical or thermal stimuli [5, 11, 12]. These hypersensitive withdrawal responses often serve as a behavioral indicator of “pain”.

Formalin, in contrast, is an aqueous solution of formaldehyde, a cell toxin that cross links proteins to disrupt dynamic protein interactions critical to cell viability. Formalin functions as an acute irritant, and when administered into the hindpaw of rodents, it elicits transient behaviors such as flinching and paw-licking [13–15, 12]. However, formalin also elicits a sustained inflammatory response characterized by edema and local release of inflammatory mediators [12]. Moreover, formalin also damages or kills cells, including primary nociceptors and other sensory neurons, and as a result, formalin-induced changes in behavior also include a neuropathic component. For example, formalin injection into the hindpaw of rodents has been shown to produce general necrosis at the site of injection, increased expression of the neuropathy-related transduction factor ATF-3 in dorsal root ganglion cell bodies, and increased spinal microglial activation to an extent similar to that produced by other neuropathy models [16, 12, 17]. Consistent with this evidence for long-lasting tissue injury, intraplantar formalin also produces hypersensitivity to mechanical and thermal stimuli, and as with CFA, this hypersensitivity is sustained for days or weeks and often serves as a behavioral indicator of pain [15, 18].

We have categorized behaviors such as hypersensitive paw withdrawal reflexes as “pain-stimulated behaviors,” which are defined as behaviors that increase in rate, frequency or intensity after delivery of a pain stimulus [1, 19]. However, pain states can also depress other behaviors, and treatment of pain-related behavioral depression is a common goal of human and veterinary medicine [20, 21]. Research on pain-related depression of behavior can be accomplished with procedures that measure “pain-depressed behaviors,” which can be defined as behaviors that decrease in rate, frequency or intensity after a pain stimulus [1, 19]. For example, intracranial self-stimulation is a procedure in which subjects perform an operant behavior (e.g. press a lever) to receive pulses of electrical stimulation that activate the mesolimbic dopamine system, and ICSS has been used to examine effects of various manipulations on brain reward function [22–24]. Previous studies have reported relatively transient depression of ICSS by acute noxious stimuli including intraperitoneal (IP) injection of dilute acid or paw incision [25–27]. The goal of the present study was to compare effects of intraplantar CFA and formalin as more sustained pain stimuli on ICSS in rats. We hypothesized that both stimuli would produce sustained depression of ICSS similar to their shared ability to produce sustained thermal and mechanical hypersensitivity. Pain-related depression of ICSS was evaluated for its sensitivity to reversal by the mu opioid analgesic morphine. In addition, pain-related depression of ICSS was evaluated for its relationship to central biomarkers of the endogenous kappa opioid system and its sensitivity to the kappa antagonist norbinaltorphimine (norBNI), because some other stressors depress behavior by activating central kappa systems [28, 29].

## Methods

### Subjects

Studies were conducted in male Sprague-Dawley rats (Harlan, Frederick MD) with initial weights of 285 to 350 g. Rats were individually housed and maintained on a 12-h light/dark cycle with lights on from 6:00 AM to 6:00 PM. Food and water were continuously available in the home cage. Animal-use protocols were approved by the Virginia Commonwealth University Institutional Animal Care and Use Committee and complied with the National Research Council (2011) Guide for the Care and Use of Laboratory Animals.

### Noxious stimuli and Drugs

CFA was obtained from Sigma Aldrich (St. Louis, MO; Catalog #F5881). Formalin was obtained from Fisher Scientific (Waltham, MA; Catalog #305-510) and diluted in saline to final concentrations as described below. Rats were lightly restrained in a soft cloth for 100 ul bilateral injections administered into the plantar aspect of the left and right hind paws using a 27 gauge needle. Morphine sulfate (National Institute on Drug Abuse Drug Supply Program; Bethesda, MD) and norbinaltorphimine 2HCl (synthesized by K. Cheng and K. Rice) were dissolved in saline for s.c. injection, and doses are expressed as the salt.

### Intracranial self-stimulation

#### Training

Sixty-eight rats were implanted with electrodes targeting the medial forebrain bundle and trained to respond for brain stimulation using experimental chambers and procedures identical to those described previously [30] *.* Briefly, a white house light was illuminated during behavioral sessions, and responding under a fixed-ratio 1 schedule produced a 0.5-s train of 0.1-ms square-wave cathodal pulses (0.1-ms pulse duration) together with 0.5 s illumination of stimulus lights over the response lever. The terminal schedule consisted of sequential 10-min components. During each component, one of a descending series of 10 current frequencies was presented (2.20-1.75 log Hz in 0.05-log increments), with a 60-s trial at each frequency. Each frequency trial consisted of a 10-s timeout, during which five noncontingent stimulations were delivered at the frequency available during that trial, followed by a 50-s “response” period, during which responding produced electrical stimulation. Training continued with presentation of three sequential components per day until rats reliably responded for the first three to six trials for all components for at least three consecutive days.

#### Experiment 1: Comparison of CFA and formalin

Once training was complete, baseline pre-injection ICSS was assessed for three consecutive days. Next, rats received bilateral intraplantar injections of CFA, 5% formalin, or saline. On the day of injection (Day 0), 3 “baseline” ICSS components were conducted before injection, and pairs of ICSS test components were conducted 1, 3, and 10 hr after injection. In addition, in the formalin and associated saline control groups, ICSS was also evaluated during five consecutive test components from 0-50 mins after injection, a time when formalin has been reported to elicit unconditioned flinching responses (“Phase I” and “Phase II” of the formalin response; [13–15, 12]. On Days 1-7 after injection, ICSS was evaluated during three-component test sessions beginning at approximately 3 PM each day. Additionally, on days 1, 3 and 7, manipulations of the height of the ICSS response lever were introduced in a subset of 6 rats from each group as a potential “use-dependent” measure of injection effects on ICSS responding. Specifically, after testing with the standard low lever height (1.5 in above the floor), ICSS was redetermined with a medium lever height (2.75 in), and a high lever height (4.5 in), and these lever heights required increasingly vertical postures by the rat and increased weight bearing by the injected rear paws. Lever heights were presented in ascending order, with a 30-min time out between testing at each height.

ICSS was significantly depressed in the formalin-treated group on Day 7 after injection. To assess the sensitivity of formalin-induced depression of ICSS to treatment with an analgesic drug, an additional test with the mu opioid agonist morphine was conducted on Day 8 in the formalin and associated control groups. Following determination of baseline ICSS performance during three ICSS components as described above, cumulative doses of morphine (0.32-3.2 mg/kg s.c.) were administered at 60 min intervals, such that each dose increased the total, cumulative dose by 0.5 log units. Thirty minutes after each dose of morphine, a pair of ICSS test components was conducted.

Paw edema, body weight and mechanical sensitivity were measured in conjunction with ICSS in this group. To assess edema, dorsal-ventral thickness of the left hind paw was measured with electronic digital calipers (Traceable Calipers, Friendswood, TX) to the nearest 0.01 mm. Body weights were assessed using an electric scale (resolution 0.1 g). The von Frey filament test was used to measure sensitivity to a punctate pressure stimulus. Rats were placed on an elevated mesh galvanized steel platform in individual chambers with a hinged lid and allowed to acclimate for at least 20 min. Subsequently, von Frey filaments (0.4 - 15 g in approximate 0.25 log increments; North Coast Medical, Morgan Hill, CA) were applied to the plantar aspect of the left hind paw using the “up-down” method to determine log median withdrawal threshold [31]. Paw thickness and body weight were assessed for each of three days before intraplantar injections and daily on Days 1-7 after intraplantar injection. Mechanical sensitivity was assessed for three days before injection, 6 hr after injection on Day 0, and on Days 3 and 7 after injection. All measurements were determined after daily ICSS sessions.

#### Experiment 2: Dose-dependence and persistence of formalin effects

Twenty-four rats were trained in the ICSS procedure and divided into four separate groups of six each that received 0.5% bilateral formalin, 5% unilateral formalin in one paw + saline in the opposite paw, 5% bilateral formalin, or bilateral saline control. ICSS was evaluated daily for three days before injection and for 14 days after injection.

#### Experiment 3: Effects of norBNI on formalin-depressed ICSS

Twelve rats were trained in the ICSS procedure and divided into two groups of six rats that received bilateral 5% formalin or bilateral saline control. ICSS was evaluated for three days before injection and for 14 days after injection. NorBNI (32 mg/kg s.c.) was administered immediately after ICSS testing on Day 7. Previous studies found that this dose of norBNI was sufficient to block depression of ICSS produced by the kappa agonist U69,593 for at least three days [30].

### Data analysis

The primary dependent measure for ICSS experiments was the total number of stimulations delivered across all 10 frequency trials of each component. The first ICSS component each day was considered to be a warm-up component, and data were discarded. Baseline ICSS in each subject was determined by averaging the number of stimulations per component during the second and third components across the three pre-injection baseline days (6 components total). Data collected after intraplantar injections for each subject were then normalized to these baselines using the equation % Baseline Stimulations per Component = (Stimulations per Test Component /Baseline) × 100.

An additional dependent measure was the reinforcement rate in stimulations/trial during each of the 10 frequency trials. To normalize these data, raw reinforcement rates from each trial in each rat were converted to percentage of maximum control rate (%MCR) for that rat, with the maximum control rate defined as the mean of the maximal rates observed during any frequency trial of the second and third baseline components across the three pre-injection baseline days. Thus, %MCR values for each trial were calculated as (response rate during a frequency trial ÷ maximum control rate) × 100.

Data for ICSS, paw thickness, body weight and mechanical sensitivity were averaged across rats in each experimental condition and compared by two- or three-way ANOVA as appropriate. For all analyses, a significant ANOVA was followed by the Holm-Sidak post-hoc test, and the criterion for significance was set a priori at *p* < 0.05.

### Quantitative real-time reverse transcriptase polymerase chain reaction

Twenty-four rats were used for qRT-PCR studies to assess CFA and formalin effects on endogenous prodynorphin (PDYN) and kappa opioid receptor (KOR) mRNA in selected brain areas as described previously [30]. Rats were treated with 100 ul bilateral intraplantar injections of saline, CFA, 0.5% formalin or 5% formalin (N = 6 per treatment), then euthanized seven days later by rapid decapitation. Brains were immediately extracted, rapidly frozen in -80°C isopentane, and stored at -80°C until analysis. Briefly, brains were sliced on a cryostat, and bilateral tissue punches were collected from ventral tegmental area, nucleus accumbens core and shell, caudate/putamen, and prefrontal cortex. PDYN and KOR mRNA values were divided by the average values of the two internal controls (β-actin and Calnexin). Values are reported as percent saline controls calculated as (normalized saline and experimental means/normalized saline group mean for the corresponding time point) × 100. Data for PDYN and KOR mRNA were analyzed using two-way ANOVA with treatment and brain areas as the two factors.

## Results

**Figure 1:**
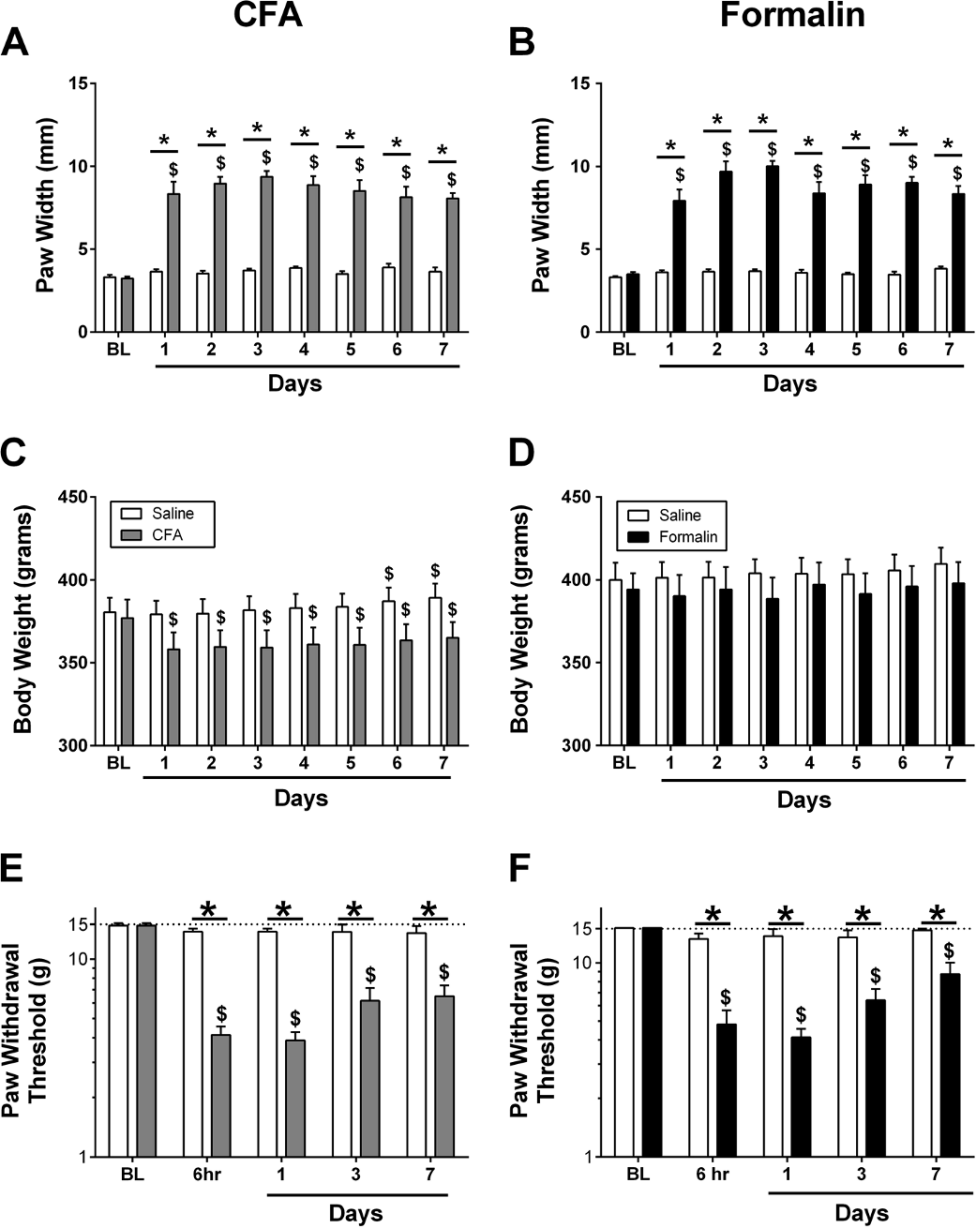
**Effects of complete Freund’s adjuvant (CFA), formalin, or respective controls on paw width, body weight and mechanical sensitivity.** The abscissae for all panels is hours or days following bilateral 100 ul injection of CFA (gray bars, Panels **A**, **C**, **E**), formalin (filled bars, Panels **B**, **D**, **F**), or respective saline controls (open bars, all panels). Bars above “BL” show baseline data before injection. Ordinates (Panels **A**, **B**): paw width in mm. Ordinates (Panels **C**, **D**): body weight in grams. Ordinates (Panels **E**, **F**): paw withdrawal threshold from von Frey filaments in grams (log scale). Dollar signs ($) indicate a significant within-group difference from the respective baseline, and asterisks (*) indicate a significant between-group difference at a given time point, as determined by a significant two-way Repeated Measures ANOVA followed by the Holm-Sidak post hoc test (*p < 0.05*). All points show mean ± SEM from 8 rats. See Additional file 1 for detailed statistical results.

Figure 1 shows effects of intraplantar saline, CFA or formalin on paw thickness, body weight and mechanical sensitivity. All statistical results for this and other figures are presented in the figure legends and in Additional file 1. Intraplantar saline had no effect on paw thickness; however, both CFA and formalin produced significant and sustained increases in paw thickness (e.g. edema) within 24 hours, and these effects persisted for 7 days (Figure 1A,B). Body weight increased significantly in the saline-control group for the CFA rats (Figure 1C), but this increase was small, and weight gain was not significant in the control group for the formalin rats (Figure 1D). Administration of CFA, but not formalin, reduced body weight within 24 hours, and body weight in CFA rats remained below baseline for 7 days (Figure 1C,D). Saline had no effect on mechanical sensitivity; however, both CFA and formalin produced significant and sustained decreases in mechanical thresholds for eliciting paw withdrawal within 6 hours, and this hypersensitivity persisted for 7 days (Figure 1E,F).

**Figure 2:**
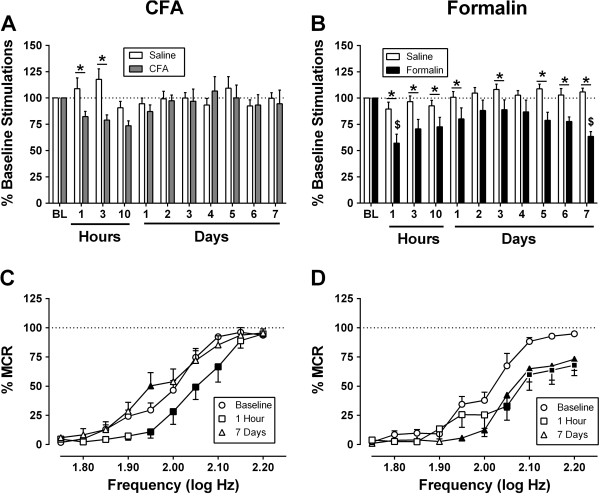
**Effects of complete Freund’s adjuvant (CFA), formalin, or respective controls on intracranial self-stimulation (ICSS).** Panels **A** and **B**: Abscissae show hours or days following bilateral 100 ul injection of CFA (gray bars, Panel **A**), formalin (filled bars, Panel **B**), or respective saline controls (open bars, both panels). Ordinates show ICSS rate expressed as total stimulations per component relative to pre-injection baseline. Dollar signs ($) indicate a significant within-group difference from the respective baseline, and asterisks (*) indicate a significant between-group difference at that time point. Panels **C** and **D** show full frequency-rate ICSS curves for selected time points from **A** and **B**. Abscissae show frequency of electrical brain stimulation (Log Hz). Ordinates show ICSS rate expressed as percent maximum control rate (%MCR). Filled points indicate statistical significance of treatment effects relative to the pre-injection baseline. All statistical analyses were performed using two-way Repeated Measures ANOVA followed by the Holm-Sidak post hoc test (*p < 0.05*). All points show mean ± SEM from 8 rats. See Additional file 1 for detailed statistical results.

Figure 2 shows effects of intraplantar saline, CFA or formalin on intracranial self-stimulation (ICSS). Prior to intraplantar injections, the mean ± SEM baseline number of stimulation per component across all stimulation frequencies was 264 ± 13, and the mean ± SEM maximum control rate (MCR) at any one frequency trial was 58.9 ± 2 stimulations per trial. Intraplantar saline treatment did not alter ICSS at any time. CFA produced weak and transient depression of ICSS. Specifically, at 1 and 3 hr after injection, ICSS in the CFA-treated rats was significantly lower than ICSS in the saline-treated controls (Figure 2A); however, ICSS in CFA- and saline-treated rats did not differ after 3 hr, and within the CFA group, ICSS after CFA treatment never differed significantly from baseline ICSS before CFA. Figure 2C shows full frequency-rate ICSS curves at selected times in the CFA-treated group. At baseline before CFA administration, electrical brain stimulation maintained a frequency-dependent increase in ICSS. In this analysis, ICSS was significantly decreased at several brain-stimulation frequencies after 1 hr but not after 7 days. In contrast to CFA, formalin produced a more robust and sustained depression of ICSS (Figure 2B). Relative to the saline control group, formalin significantly reduced ICSS during the first 24 hr after treatment and again during Days 5-7 after treatment. Relative to the pre-formalin baseline within the formalin group, formalin significantly depressed ICSS during the first 10 hr and again on Day 7 after treatment. Figure 2D shows full frequency-rate curves at selected times in the formalin treated group. Formalin significantly depressed ICSS at multiple frequencies both 1 hr and 7 days after treatment.

Additional file 2: Figure S1 shows formalin effects on ICSS during the first 50 min after formalin administration. Formalin decreased ICSS throughout the first 50 min of testing, although the magnitude of this decrease was greatest from 20-50 min after treatment.

Additional file 3: Figure S2 shows CFA and formalin effects on ICSS at different lever heights in a subset of six rats from each group on Days 1, 3 and 7 after intraplantar treatment. In general, ICSS decreased as lever height increased, and this effect was largest on Day 1. CFA failed to significantly alter ICSS at any lever height on any day, whereas formalin depressed ICSS across all lever heights and days.

**Figure 3:**
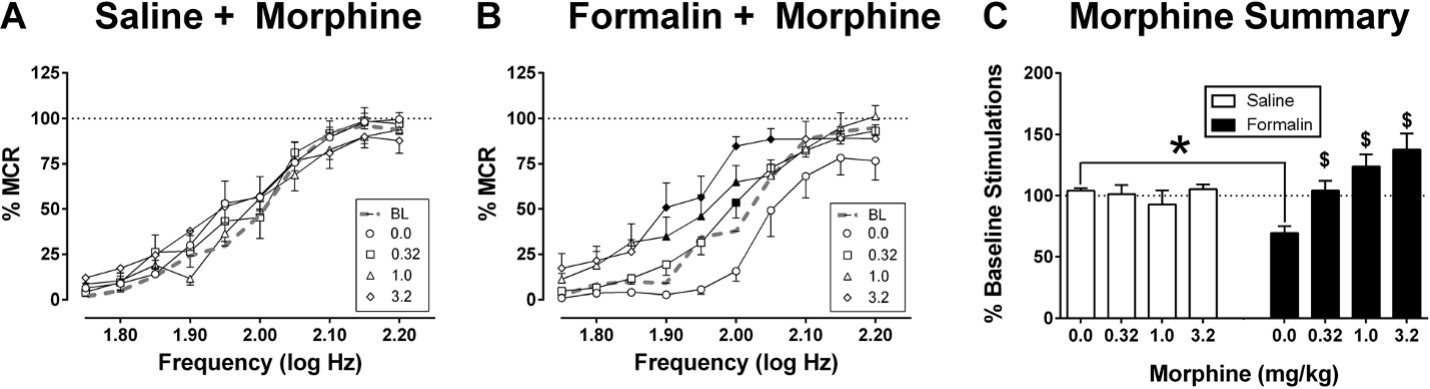
**Effects of the mu opioid agonist morphine (0.32-3.2 mg/kg) on ICSS eight days after bilateral intraplantar saline or 5% formalin.** Panels **A** and **B**: Abscissae show frequency of electrical brain stimulation (Log Hz) in rats that received intraplantar saline **(A)** or formalin **(B)**. Ordinates show ICSS rate expressed as percent maximum control rate (%MCR). “BL” shows the baseline frequency-rate curve determined before intraplantar treatment, “0.0” shows the frequency-rate curve determined on Day 8 after intraplantar treatment but before morphine treatment. Filled points in panel **B** show significant morphine effects relative to “0.0.” Panel **C**: The abscissa shows morphine dose in mg/kg in rats that received intraplantar saline (open bars) or formalin (filled bars). The ordinate shows ICSS rate expressed as total stimulations per component relative to baseline. Dollar signs ($) indicate a significant within-group difference from “0.0”, and asterisks (*) indicate a significant between-group difference at a given morphine dose. All statistical analyses were performed using a two-way Repeated Measures ANOVA followed by the Holm-Sidak post-hoc test (*p < 0.05*). All data show mean ± SEM from 8 rats per treatment. See Additional file 1 for detailed statistical results.

Figure 3 shows effects of morphine on ICSS 8 days after treatment with intraplantar saline or formalin. In the saline-treated rats, cumulative dosing with 0.32-3.2 mg/kg morphine produced no significant effect on ICSS. Conversely, in the formalin-treated rats, baseline ICSS was depressed before morphine administration, and morphine produced a dose-dependent reversal of formalin-induced depression of ICSS. The lowest dose of 0.32 mg/kg morphine was sufficient to restore ICSS back to approximately baseline levels, and higher morphine doses produced a further facilitation of ICSS.

**Figure 4:**
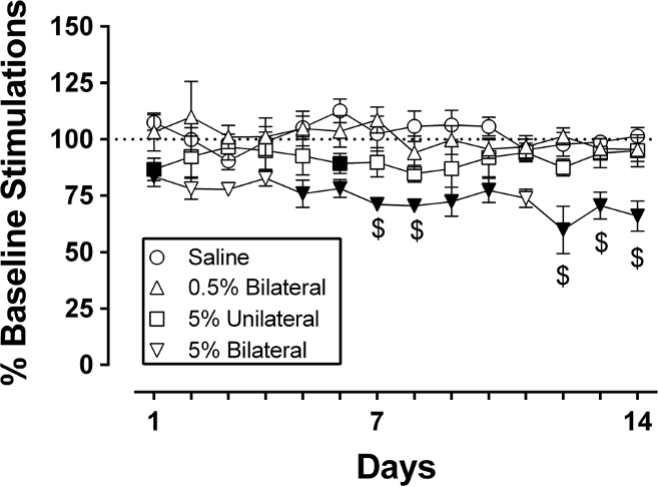
**Formalin induced-depression of intracranial self-stimulation (ICSS) is dose and time related.** Abscissa: Days after varying doses of intraplantar formalin or saline administration. Ordinate: ICSS rate expressed as percent baseline stimulations per component. Statistical analysis was performed using two-way Repeated Measures ANOVA followed by the Holm-Sidak post hoc test (*p < 0.05*). Dollar signs ($) indicate a significant within-group difference from the pre-injection baseline, and filled points indicate a significant between-group difference at that time point relative to saline treatment. All data show mean ± SEM from 6 rats per treatment. See Additional file 1 for detailed statistical results.

Figure 4 compares ICSS performance over a 14-day period after treatment with (a) bilateral saline, (b) bilateral 0.5% formalin, (c) unilateral 5% formalin in one paw + unilateral saline in the other paw, and (d) bilateral 5% formalin. Prior to intraplantar injections, the mean ± SEM baseline number of stimulations per component across all stimulation frequencies was 274 ± 14, and the mean ± SEM MCR at any one frequency was 55.9 ± 3. As in the first experiment, bilateral 5% formalin depressed ICSS relative to bilateral saline treatment, and in this second experiment, the formalin effect persisted for 14 days. Unilateral 5% formalin decreased ICSS relative to saline controls only on Days 1 and 6, and bilateral 0.5% formalin did not alter ICSS relative to the saline controls.

**Figure 5:**
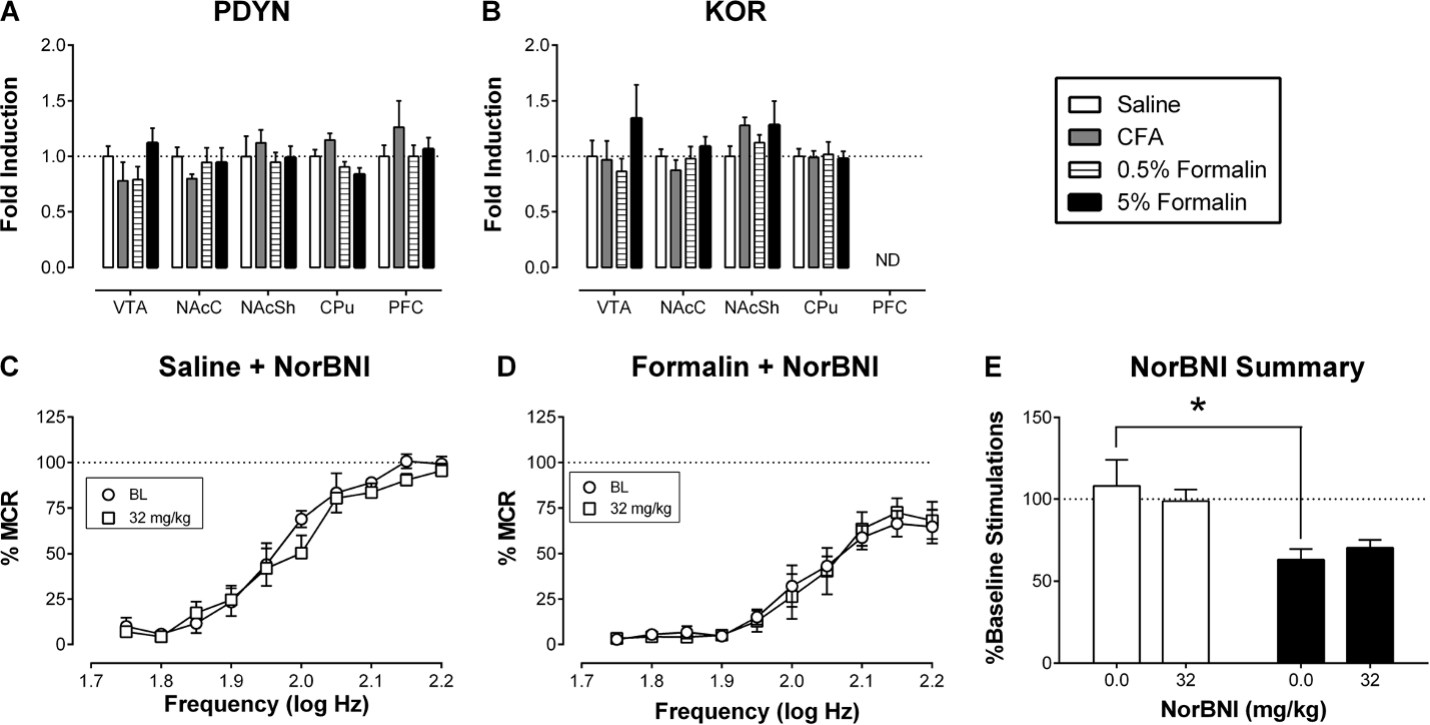
**Role of the endogenous kappa opioid system in pain-related depression of ICSS.** Panels **A** and **B**: Transcript levels of PDYN **(A)** or KOR **(B)** mRNA as measured by qRT-PCR in brain regions implicated in mood disorders (CPu: caudate/putamen, NAc: nucleus acumbens core, NAcSh: nucleus acumbens shell, PFC: prefrontal cortex, VTA: ventral tegmental area). Abscissae: Brain area evaluated. Ordinates: Transcript levels expressed as “Fold-Induction” relative to saline controls. “ND” in Panel **B** signifies “Not Determined” due to low transcript levels below the level of detection in some rats. Panel **C-D**: Effects of the kappa antagonist norBNI (32 mg/kg) on ICSS in rats treated with intraplantar saline **(C)** or formalin **(D)**. Abscissae show frequency of electrical brain stimulation (Log Hz). Ordinates show ICSS rate expressed as percent maximum control rate (%MCR). “BL” shows the frequency-rate curve determined on Day 7 after intraplantar saline or formalin and immediately before norBNI treatment. ICSS was then redetermined 24 hr after norBNI. Summary data are shown in Panel **E**, where the abscissa shows norBNI dose in mg/kg in rats that received intraplantar saline (open bars) or formalin (filled bars), and the ordinate shows ICSS rate expressed as total stimulations per component relative to the pre-formalin baseline. The asterisk (*) indicates a significant between-group difference at a given dose. All data show mean ± SEM from 6 rats. See Additional file 1 for detailed statistical results.

Figure 5 shows data to address the role of kappa opioid systems in mediating effects of CFA and formalin on ICSS. Neither CFA nor formalin significantly altered expression of PDYN or KOR mRNA in any brain area examined on Day 7 after intraplantar treatment (Figure 5A and B). Moreover, the kappa antagonist norBNI failed to alter ICSS in rats treated with intraplantar saline, and it also failed to block formalin-induced depression of ICSS in rats treated with intraplantar formalin (Figure 5C-E). Data in Figure 5C-E show results obtained on Day 8 after intraplantar injection and 22 hr after norBNI administration. Rats were also tested daily for another six days (Days 9-14 after intraplantar injection), and norBNI did not significantly alter either control ICSS or formalin-depressed ICSS on any day (data not shown).

## Discussion

This study compared effects of intraplantar CFA and formalin on a series of behavioral and physiological endpoints in rats. There were four main findings. First, in agreement with previous studies, both CFA and formalin produced similar degrees of paw swelling and mechanical hypersensitivity. Second, CFA and formalin produced different effects on ICSS. Specifically, CFA produced relatively weak and transient depression of ICSS, whereas formalin produced a more robust and sustained depression of ICSS that lasted as long as 14 days. Third, formalin-induced depression of ICSS could be reversed by morphine doses that had no significant effect on ICSS in saline-treated rats, suggesting that formalin effects on ICSS can be interpreted as an example of pain-related and analgesic-reversible depression of behavior. Finally, neither behavioral nor central biomarker data support a role for the endogenous kappa opioid system in mediation of formalin-induced depression of ICSS. These results suggest differential efficacy of sustained pain stimuli to depress brain reward function in rats as assessed with ICSS.

### CFA-and formalin effects on paw width, mechanical allodynia and body weight

The CFA and formalin effects reported here agree with previous studies in rats that examined the time course of paw swelling and/or mechanical sensitivity after intraplantar CFA [5, 31, 18] or formalin [15, 32, 18]. For example, Fu et al. [15] demonstrated that a 5% formalin injection into the hindpaw of rats produced both mechanical and thermal allodynia for up to four weeks following administration. Similarly, Grace et al. [18] found that bilateral injection of either CFA or formalin into the hindpaw resulted in mechanical allodynia that lasted up to seven days. Transient weight loss in CFA-treated rats, but not formalin-treated rats, is also consistent with previous studies. For example, 100 μl CFA administered to the tail-base in rats produced a magnitude and time course of weight loss similar to that reported here [33], whereas rats gained weight normally for six weeks after unilateral intraplantar injection of 50 μl 5% formalin [34].

### Differential effects of CFA and formalin on ICSS

Although CFA and formalin produced similar effects on mechanical allodynia as a measure of pain-stimulated behavior, they produced distinct effects on depression of ICSS as a measure of pain-depressed behavior. The greater and more sustained efficacy of formalin to depress ICSS may be related to its induction of necrosis in the paw, neuropathy of primary afferents, and/or microglial activation at the level of the spinal cord [16, 12, 17], and we are actively investigating the role of these formalin effects in formalin-induced depression of ICSS. However, regardless of mechanism, these results extend the range of pain-related stimuli that have been found to depress brain reward function as assessed with ICSS in rats, and further identify bilateral intraplantar formalin as the stimulus producing the most sustained depression of ICSS so far reported. For example, previous studies have shown transient (1-2 hr) pain-related and analgesic-reversible depression of ICSS by intraperitoneal injection of dilute acid [25, 26], and ICSS was also depressed for up to three hours by intraplantar CFA (present study) and for up to two days by paw incision [27]. In contrast, effects of bilateral intraplantar formalin in the present study lasted for at least 14 days. Moreover, the poor efficacy of unilateral intraplantar formalin to alter ICSS in this study agrees with the finding that a unilateral spinal nerve ligation-model of neuropathy also failed to alter ICSS at any time [27].

The present evaluation of CFA and formalin effects on ICSS also warrant comparison to CFA and formalin effects on some other metrics of pain-related behavioral depression and/or negative affective states. For example, unilateral treatment in rats with intraplantar CFA doses similar to that used here depressed diurnal exploratory activity for four weeks [35] and burrowing for 10 days [36]; however, pain-related changes in facial expression or place conditioning were apparent for only one day [37, 38], and neither nocturnal locomotor activity nor wheel running were significantly affected at any time [35, 18]. Bilateral CFA injection, such as that used in the present study, did depress both nocturnal locomotor activity (for four weeks) and wheel running (for two days) in rats, and studies in mice have also reported a requirement for bilateral CFA treatment to produce transient depression of wheel running [39]. Taken together, these results indicate that CFA has different efficacies and time courses to produce different pain-related behaviors, and ICSS in rats is relatively resistant to CFA effects.

Fewer studies have examined effects of formalin in procedures of pain-related behavioral depression and/or negative affective states. Perhaps of greatest relevance to the present study, bilateral intraplantar formalin produced avoidance for six weeks of noxious thermal stimuli in an operant-escape procedure [34]. Intraplantar formalin has also been shown to produce pain-related changes in facial expression and place conditioning [40–42], but these effects were evaluated only for the first hour after formalin administration, and more sustained formalin effects on these procedures have not been examined. Lastly, in contrast to formalin effects on ICSS, bilateral intraplantar formalin administration had no effect on wheel running in rats [18]. This distinction is notable, because the failure of bilateral intraplantar formalin to alter either body weight (present study) or wheel running [18] provides evidence to suggest that ICSS depression by formalin could not be attributed to general behavioral impairment.

### Morphine reversal of formalin-induced depression of ICSS

The failure of morphine to significantly alter ICSS in rats after intraplantar saline treatment is consistent with previous studies showing little or no effect of these morphine doses on ICSS in opioid-naïve rats [25, 43, 44]. However, these same morphine doses significantly reversed formalin-induced depression of ICSS, consistent with previous studies showing that morphine also blocks acute depression of ICSS by IP acid [25, 19]. Moreover, the high potency of morphine to block formalin-induced depression of ICSS (effective at 0.32 mg/kg) is similar to the high potency of morphine to block acid-induced depression of ICSS [25]. Reversal of formalin-induced depression of ICSS by the opioid analgesic morphine provides one source of evidence to suggest that this formalin effect may be related to sustained pain.

In the present study, high morphine doses not only reversed formalin-induced depression of ICSS but also increased ICSS above original baseline levels. Mechanisms responsible for this morphine effect are not currently known; however, the emergence of rate-increasing effects produced by these morphine doses after formalin treatment is similar to the emergence or enhancement of rate-increasing effects produced by regimens of prior morphine exposure [45]. Formalin treatment has been reported to promote endogenous opioid release [46–48], and this raises the possibility that endogenous opioid release stimulated by formalin treatment had the effect of sensitizing rats to rate-increasing effects of subsequent treatment with the exogenous opioid morphine.

### NorBNI failed to reverse formalin-induced depression of ICSS

Administration of the endogenous kappa agonist dynorphin or of exogenous kappa agonists like salvinorin A is sufficient to decrease mesolimbic dopamine release and to depress ICSS in rodents [49–51, 43]. In addition, previous studies have shown that some non-pain stressors can increase central biomarkers for kappa opioid function and produce depression-like behaviors that can be blocked by kappa antagonists [52, 53, 29, 54]. These findings have suggested the possibility that activation of endogenous kappa opioid systems might also mediate pain-related depression of ICSS. Accordingly, the present study tested the hypothesis that CFA and/or formalin might activate endogenous kappa opioid signaling and produce kappa antagonist-reversible depression of ICSS. However, the present results do not support this hypothesis for four reasons. First, neither CFA nor formalin significantly increased central PDYN or KOR mRNA levels. Second, although this analysis may have failed to detect small but real changes in kappa biomarkers (a Type II error), there was no pattern for either a trend toward increased biomarker levels or a difference in CFA and formalin effects on biomarkers consistent with the difference in their effects on ICSS. Third, CFA- and formalin-induced changes in PDYN never approached the nearly two-fold increase in PDYN produced in rats exposed to the stress of a forced swim test [53]. Finally, the formalin-induced decrease in ICSS was not blocked by the kappa antagonist norBNI, suggesting that any modest effects that formalin might have had on kappa biomarkers were not sufficient to produce a kappa receptor-mediated decrease in ICSS.

## Electronic supplementary material

Additional file 1: **Detailed Statistical Results.** (DOC 26 KB)

Additional file 2: Figure S1: Shows effects of formalin (filled bars) or saline (open bars) on ICSS during the first 50 minutes of testing immediately following intraplantar administration. Abscissa: Time (in 10 minute bins) after intraplantar formalin or saline administration. Ordinates: ICSS rate expressed as total stimulations per component relative to baseline. Data were analyzed by two-way ANOVA followed by the Holm-Sidak post-hoc test (*p < 0.05*). All points show mean ± SEM from 8 rats. Statistical results are as follows. Significant main effect of treatment [F(1, 14) = 13.141; p = 0.003], significant main effect of time [F(4,56) = 13.394; p < 0.001], and a significant interaction of treatment x time [F(4,56) = 3.928; p = 0.007]. Dollar signs ($) indicate a significant within-group difference from the respective baseline, and asterisks (*) indicate a significant between-group difference at a given time point, as determined by a significant two-way ANOVA followed by the Holm-Sidak post hoc test (*p < 0.05*). (JPEG 1 MB)

Additional file 3: Figure S2: Shows effects of complete Freund’s adjuvant (CFA, filled gray bars in Panel **A**), formalin (filled bars in Panel **B**), or respective controls (open bars in both panels) on ICSS during lever height challenges. Abscissae show lever heights (low, middle or high) on days 1, 3 and 7 following treatment. Ordinates show ICSS rate expressed as total stimulations per component relative to baseline determined at the low lever height before intraplantar treatment. Data were analyzed by three-way ANOVA followed by the Holm-Sidak post-hoc test (*p < 0.05*). All points show mean ± SEM from 6 rats. Statistical results are as follows. Panel **A**. Significant main effect of day [F(2,90) = 4.148, p = 0.019], significant main effect of lever height [F(2,90) = 9.097, p < 0.001], but no main effect of CFA treatment [F(1,90) = 0.121, p = 0.729] and no significant interactions of day X lever height (p = 0.785), day X treatment (p = 0.417), lever height X treatment (p = 0.696), or day X lever height X treatment (p = 0.803). Panel **B**. Significant main effect of day [F(2,90) = 4.669, p = 0.012], significant main effect of lever height [F(2,90) = 27.314, p < 0.001], and a significant main effect of formalin treatment [F(1,90) = 78.725, p < 0.001], but no interactions of day X lever height (p = 0.108), day X treatment (p = 0.154), lever height X treatment (p = 0.794), or day X lever height X treatment (p = 0.558). Panels C-E show the posture of a rat responding at the low, medium and high lever height, respectively. Increases in lever height required increasingly erect postures and increased weight bearing on the hind paws. (JPEG 3 MB)
