# Supplementary material for: Sustained pain-related depression of behavior: effects of intraplantar formalin and complete freund’s adjuvant on intracranial self-stimulation (ICSS) and endogenous kappa opioid biomarkers in rats
Source: Mol Pain. 2014 Sep 23;10:62. doi: 10.1186/1744-8069-10-62 (PMC4180532; doi:10.1186/1744-8069-10-62)
Supplement: Supplementary file 1 — Additional file 1: Detailed Statistical Results. (DOC 26 KB) [file 12990_2014_666_MOESM1_ESM.doc]

**Additional file 1**

**Detailed Statistical Results:**

**Figure 1:** Statistical results are as follows. Panel A. Significant main effects of treatment [F(1,14) = 123.210, p < 0.001] and time [F(7,98) = 27.595, p < 0.001], and a significant interaction [F(7,98) = 21.226, p < 0.001]. Panel B. Significant main effect of treatment [F(1,14) = 219.110, p < 0.0.001], and time [F(7,98) = 21.906, p < 0.001], and a significant interaction [F(7,98) = 18.013, p < 0.001]. Panel C. No significant main effect of treatment [F(1,14) = 2.321, p = 0.150], but a significant main effect of time [F(7,98) = 13.822, p < 0.001], and a significant interaction between treatment and time [F(7,98) = 11.912, p < 0.001]. Panel D. No significant main effect of treatment [F(1,14) = 0.050, p = 0.826], a significant main effect of time [F(7,98) = 2.673, p = 0.014], and no significant interaction [F(7,98) = 0.513, p = 0.821]. Panel E. Significant main effect of treatment [F(1,14) = 100.807, p < 0.001] and time [F(4,56) = 22.014, p < 0.001], and a significant interaction [F(4,56) = 17.253, p < 0.001]. Panel F. Significant main effect of treatment [F(1,14) = 106.624, p < 0.0.001], and time [F(4,56) = 17.067, p < 0.001], and a significant interaction [F(4,56) = 10.463, p < 0.001].

**Figure 2:** Statistical results are as follows. Panel **A**. No significant main effect of treatment [F(1,14) = 1.862, p = 0.194], no significant main effect of time [F(10,140) = 1.382, p = 0.194], but a significant interaction [F(10,140) = 1.994, p = 0.038]. Panel **B**. Significant main effect of treatment [F(1,14) = 9.860, p < 0.007] and time [F(10,140) = 5.584, p < 0.001], and a significant interaction [F(10,140) = 2.499, p = 0.009]. Panel **C**. Significant main effect of frequency [F(9,63) = 119.2, p < 0.001] and treatment [F(2,14) = 4.2123, p = 0.0369], and a significant interaction [F(18,126) = 1.722, p = 0.0434]. Panel **D**. Significant main effect of frequency [F(9,63) = 67.78, p < 0.001] and treatment [F(2,14) = 4.072, p < 0.0404], and a significant interaction [F(18,126) = 1.888, p = 0.0224].

**Figure 3:** Statistical results are as follows. Panel A. Significant main effect of frequency [F(9,63) = 197.9, p < 0.0001], but not dose [F(3,21) = 0.2735, p = 0.0.8438] and no significant interaction [F(27,189) = 0.8897, p = 0.6261]. Panel B. Significant main effect of frequency [F(9,63) = 91.60, p < 0.0001], dose [F(3,21) = 9.867, p < 0.0003], and a significant interaction [F(27, 189) = 1.875, p = 0.0083]. Panel C. No significant main effect of formalin treatment [F(1,14) = 1.1664, p = 0.2180], a significant main effect of morphine dose [F(3,42) = 5.788, p = 0.0021], and a significant interaction [F(3,42) = 6.949, p < 0.0007].

**Figure 4:** Statistical results are as follows. Significant main effect of treatment [F(3,20) = 21.23, p < 0.001], but no main effect of day [F(13,260) = 0.9393, p = 0.760] and no significant interaction [F(39,260) = 0.7640, p = 0.8436].

**Figure 5:** Statistical results are as follows. All statistical analyses were performed using a two-way ANOVA followed by the Holm-Sidak post-hoc test (p < 0.05). A two-way Repeated Measures ANOVA followed by the Holm-Sidak post-hoc test (p < 0.05) was used for all within group comparisons (panels C,D,E) Statistical results are as follows: Panel A. No main effect of brain area [F(4,94) = 1.303, p = 0.2746] or treatment [F(3,94) = 0.7809, p = 0.5075], and the interaction was also not significant [F(12,94) = 1.213, p = 0.2856], Panel **B**. No main effect of brain area [F(3,78) = 1.722, p = 0.1693], or treatment [F(3,78) = 0.1.691, p = 0.1759], and the interaction was not significant [F(9,78) = 0.8032, p = 0.6145], Panel C. Main effect of frequency [F(9,45) = 110.1, p < 0.0001], no main effect of treatment [F(1,5) = 0.001, p = 0.7754], and no interaction [F(9,45) = 0.4537, p = 0.8973]. Panel **D**. Main effect of frequency [F(9, 45) = 34.49, p < 0.0001], no main effect of treatment [F(1, 5) = 0.001, p = 0.9969], and no interaction [F(9,45) = 0.2372, p = 0.8970]. Panel E. Main effect of formalin treatment [F(1, 10) = 9.578, p = 0.0.0113], no main effect of norBNI dose [F(1, 10) = 0.02292, p = 0.8827], and no interaction [F(1,10) = 1.517, p = 0.2462].
