# Supplementary figures and images for: Sustained pain-related depression of behavior: effects of intraplantar formalin and complete freund’s adjuvant on intracranial self-stimulation (ICSS) and endogenous kappa opioid biomarkers in rats
Source: Mol Pain. 2014 Sep 23;10:62. doi: 10.1186/1744-8069-10-62 (PMC4180532; doi:10.1186/1744-8069-10-62)

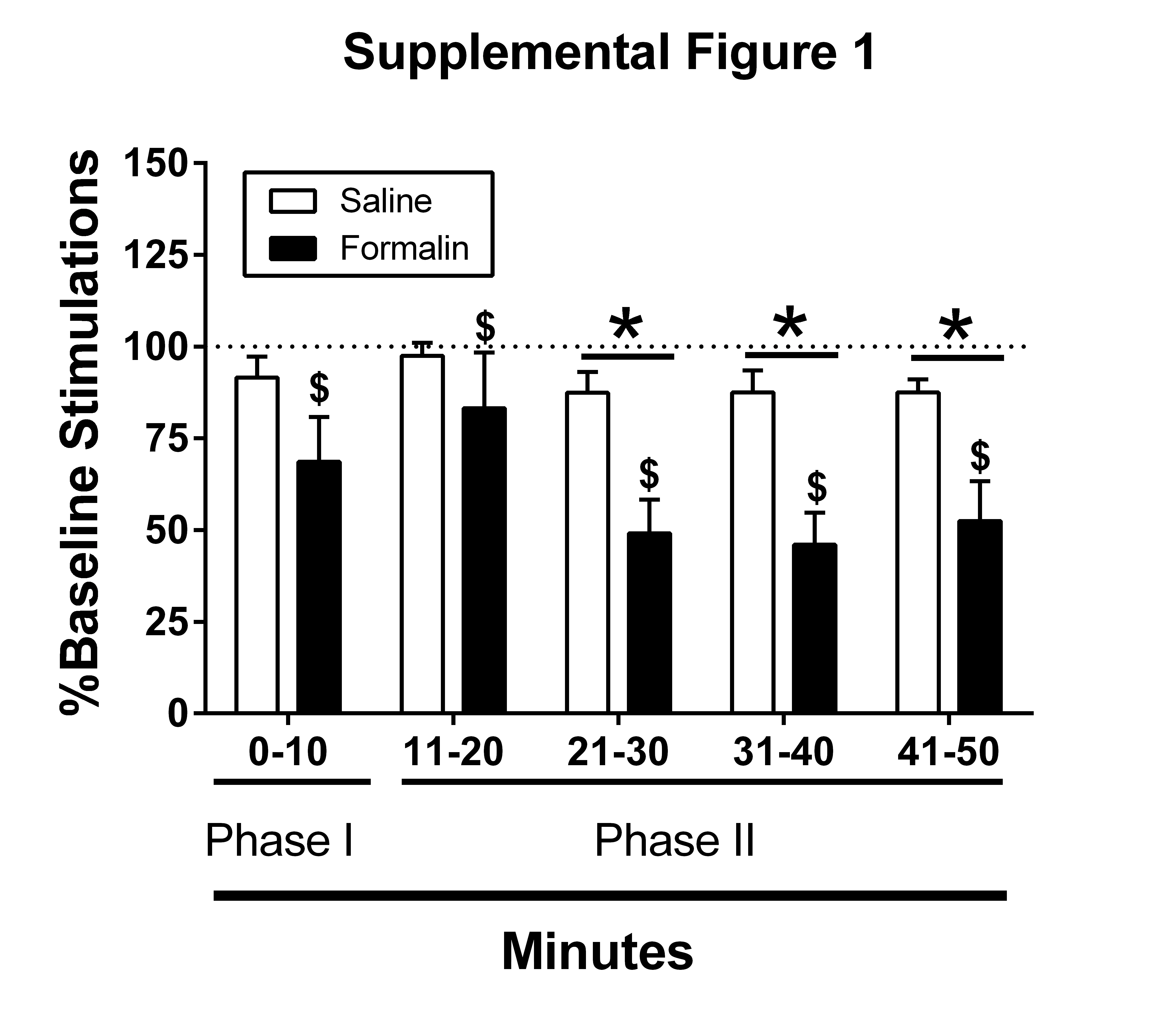

Supplement: Supplementary file 2 — Additional file 2: Figure S1: Shows effects of formalin (filled bars) or saline (open bars) on ICSS during the first 50 minutes of testing immediately following intraplantar administration. Abscissa: Time (in 10 minute bins) after intraplantar formalin or saline administration. Ordinates: ICSS rate expressed as total stimulations per component relative to baseline. Data were analyzed by two-way ANOVA followed by the Holm-Sidak post-hoc test (p < 0.05). All points show mean ± SEM from 8 rats. Statistical results are as follows. Significant main effect of treatment [F(1, 14) = 13.141; p = 0.003], significant main effect of time [F(4,56) = 13.394; p < 0.001], and a significant interaction of treatment x time [F(4,56) = 3.928; p = 0.007]. Dollar signs ($) indicate a significant within-group difference from the respective baseline, and asterisks (*) indicate a significant between-group difference at a given time point, as determined by a significant two-way ANOVA followed by the Holm-Sidak post hoc test (p < 0.05). (JPEG 1 MB) [file 12990_2014_666_MOESM2_ESM.jpeg]

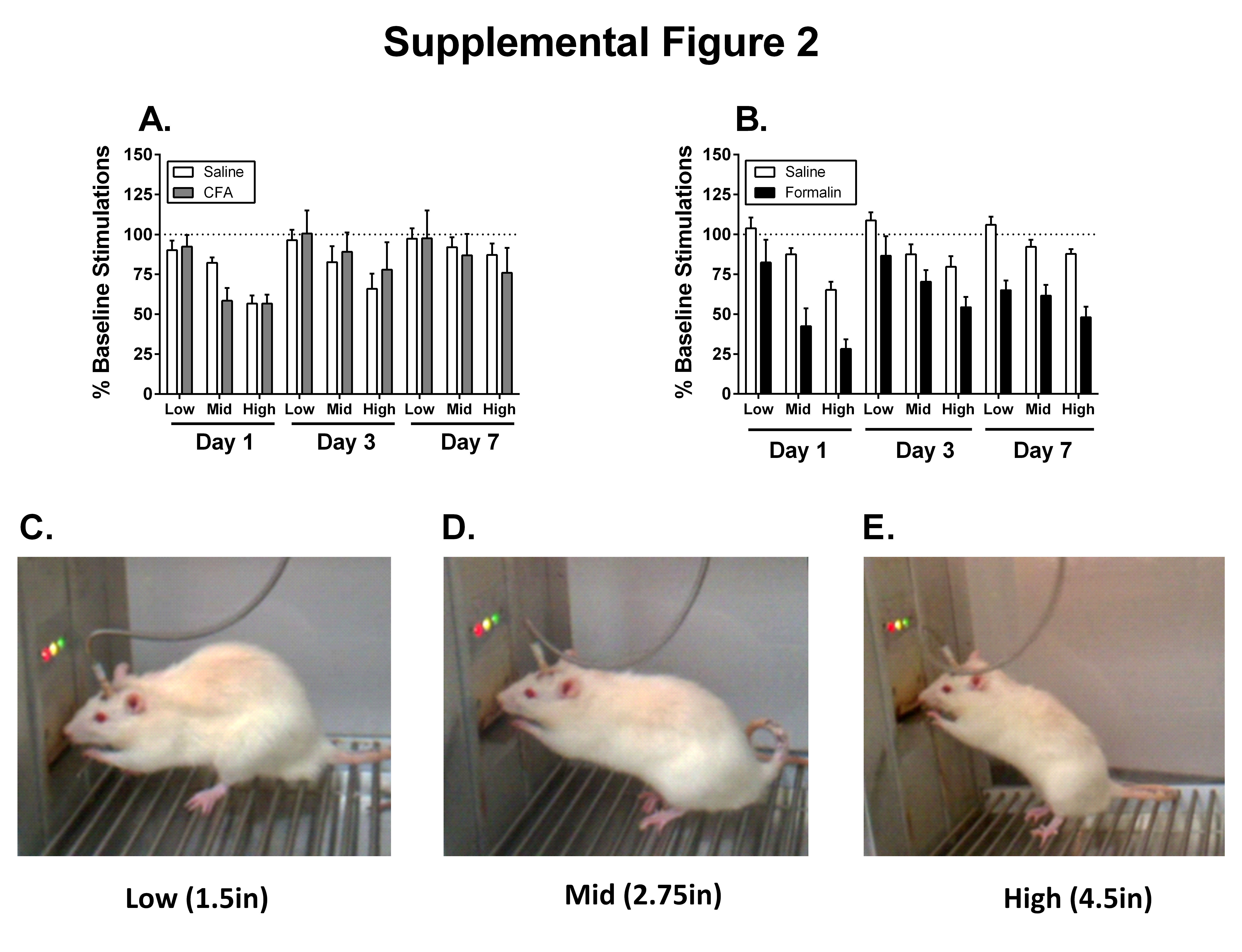

Supplement: Supplementary file 3 — Additional file 3: Figure S2: Shows effects of complete Freund’s adjuvant (CFA, filled gray bars in Panel A), formalin (filled bars in Panel B), or respective controls (open bars in both panels) on ICSS during lever height challenges. Abscissae show lever heights (low, middle or high) on days 1, 3 and 7 following treatment. Ordinates show ICSS rate expressed as total stimulations per component relative to baseline determined at the low lever height before intraplantar treatment. Data were analyzed by three-way ANOVA followed by the Holm-Sidak post-hoc test (p < 0.05). All points show mean ± SEM from 6 rats. Statistical results are as follows. Panel A. Significant main effect of day [F(2,90) = 4.148, p = 0.019], significant main effect of lever height [F(2,90) = 9.097, p < 0.001], but no main effect of CFA treatment [F(1,90) = 0.121, p = 0.729] and no significant interactions of day X lever height (p = 0.785), day X treatment (p = 0.417), lever height X treatment (p = 0.696), or day X lever height X treatment (p = 0.803). Panel B. Significant main effect of day [F(2,90) = 4.669, p = 0.012], significant main effect of lever height [F(2,90) = 27.314, p < 0.001], and a significant main effect of formalin treatment [F(1,90) = 78.725, p < 0.001], but no interactions of day X lever height (p = 0.108), day X treatment (p = 0.154), lever height X treatment (p = 0.794), or day X lever height X treatment (p = 0.558). Panels C-E show the posture of a rat responding at the low, medium and high lever height, respectively. Increases in lever height required increasingly erect postures and increased weight bearing on the hind paws. (JPEG 3 MB) [file 12990_2014_666_MOESM3_ESM.jpeg]
